# Sutureless Total Thyroidectomy for Substernal Goiter: Amending Versus Unnecessary

**DOI:** 10.7759/cureus.12720

**Published:** 2021-01-15

**Authors:** Ismail Aydin, Ilker Sengul, Demet Sengul

**Affiliations:** 1 General Surgery, Giresun University Faculty of Medicine, Giresun, TUR; 2 Endocrine Surgery, General Surgery, Giresun University Faculty of Medicine, Giresun, TUR; 3 Pathology, Giresun University Faculty of Medicine, Giresun, TUR

**Keywords:** indeterminate cytology, thyroid, substernal goiter, pathology, thyroidectomy, sutureless thyroidectomy, ligament of berry, recurrent laryngeal nerve, ligasure, thyroidology

## Abstract

Substernal goiter is an enlarged thyroid gland, harboring a component extending into the mediastinum. Surgical management requires genuine and rigorous preoperative planning as physicians could encounter the prospect of the gland coming into close quarters with the vital intrathoracic structures. The neck and chest multiplanar cross-sectional imaging provide essentialness of an extracervical approach for the procedure. In the present study, a 57-year-old female who admitted with the intermittent dyspnea and dysphagia with a huge goiter is reported. The labs were reported within the normal limits and the video laryngoscopy displayed no pathologic finding. Her neck sonography revealed the multiple nodules within the gland, without determining the most proximal border of the left lobe. The neck and chest computed tomography depicted a substernal goiter harboring the left lobe, extending till the left innominate vein and a sutureless total thyroidectomy by the collar incision without a median sternotomy was performed. We would recommend sutureless thyroidectomy for substernal goiter just considering to divide meticulously the superior thyroid arteries and veins separately and exploring the fibrous Ligament of Berry, that is, the true Ligament of Berry, with its safe relationship to the recurrent laryngeal nerve in Thyroidology.

## Introduction

Since first being described by Haller in 1749, substernal goiter is simply defined as an enlargement of the thyroid gland with a component extending to the mediastinum. The surgical approach should be planned meticulously, particularly in order for proximity to the intrathoracic vital structures in topographic anatomy [1]. Substernal goiter is usually involved within the anterior mediastinum while accounting for 10%-15% within the posterior one. The surgical approach is a curative treatment in the case of symptomatic substernal goiter, preventing potentially fatal symptoms of compressions [2]. Fundamentally, the way of surgical approach to a substernal goiter is principally decided based on the availability of easy and appropriate access to the mediastinum, considering the vital organs on the basis of the findings of relevant multiplanar cross-sectional imaging(s). Recent advances in thyroid surgery have provided using the vascular sealing systems instead of traditionally made knots [3].

Given that a vast majority of substernal goiter can be safely removed transcervical, entailment of a cardiothoracic back up should be evaluated preoperatively. LigaSure Small Jaw (LSJ) (LF1212A) uses a combination of energy and pressure application for the procedure of vessel and tissue fusion and seals the vessels and tissue bundles permanently up to seven mm in diameter without requiring any dissection and isolation. The seals can withstand up to three times the normal systolic pressure. The average seal cycle is 2-4 seconds and the feedback-controlled response system automatically de-energizes as the seal cycle is complete, without requiring the relevant assumption and coagulation occurs when collagen and elastin are denatured to form a seal [3]. In the present study, it is purposed to present an LSJ-assisted sutureless thyroidectomy for the case with substernal goiter.

## Technical report

A 57-year-old female admitted with a month history of intermittent dyspnea and dysphagia last months. Her personal medical history was unremarkable, without any previous systemic diseases or operation, and her vital signs were recorded within the normal limits. A physical examination at the time of admission revealed a huge goiter and the neck sonography revealed the multiple nodules, the larger of which in the left lobe without able to detect its most proximal border. Her labs were reported within the normal limits in terms of the thyroid function tests and the video laryngoscopy revealed no pathologic finding. The neck and chest computed tomography revealed a substernal goiter harboring the left lobe extending till the left innominate vein, also known as the left brachiocephalic vein. A total thyroidectomy by the collar incision without a median sternotomy was decided owing to facing with a symptomatic substernal goiter, comprising multiple nodules bilaterally, that did not extend beyond the left innominate vein proximally, such as the arcus aorta.

The informed consent was received for both her treatment and the present article, preoperatively. She was brought to the operating room and placed on the operating table in the supine position. Afterwards, the relevant total thyroidectomy was performed, compatible with Iowa Head and Neck Protocols [4]. Herein, she was then transorally intubated and a skin crease incision planned at one fingerbreadth above the clavicles. A shoulder roll was placed and the neck was prepared with chlorhexidine and draped in a sterile fashion. After administrating the lidocaine 1% and epinephrine 1:100,000 into the incision line, a 15-blade knife was used to perform the planned Kocher's transverse collar incision, which was carried down through the platysma to the level of the strap muscles of the neck. The subplatysmal flaps were then elevated, the strap muscles were split in the midline raphae, and the thyroid gland was explored. In dissecting around the left superior pole of the thyroid, the left superior parathyroid gland was identified and was dissected away from the left thyroid lobe. The gland was then retracted superiorly and laterally in order of releasing the left inferior pole. The left inferior parathyroid gland was identified and preserved and then the left lobe was retracted out of the surgical bed by releasing its remaining attachments of the thyroid gland from the trachea. The gland was carefully examined in order of being convinced for not harboring any evidence of parathyroid tissue on it after the removal process. Subsequently, the right side was dissected and spared in a similar fashion with the relevant identifications of the right superior parathyroid gland and right recurrent laryngeal nerve (RLN). After both thyroid lobes with the isthmus and pyramidal lobe were removed with the aid of the LSJ, the relevant haemostasis was provided and the wound was profusely and meticulously irrigated with the sterile saline. No evidence of any lymphadenopathy in the level VI, cervical compartment, was notified. A 15-French Jackson-Pratt drain was inserted and secured to the skin with 3-0 non-absorbable suture. The wound was then closed by reapproximating the strap muscles and platysma with deep 3-0 vicryl stitches and the skin by a running 4-0 monocryl subcuticular continuous stitch with the benzoin and steri-strips, placing over the incision. Consequently, the patient had tolerated the procedure well, was then extubated in the operating room uneventfully and subsequently transferred to the post-anesthesia care unit. No intra-, peri-, and postoperative complications and morbidity have developed, she demonstrated the improved symptoms and discharged home on the hospital day 1.

## Discussion

Even though various definitions have propounded for substernal goiter, it is commonly defined as a thyroid mass that extends three or more centimeters below the suprasternal notch while the neck is in the hyperextended condition. That extension might accounts for 1%-20% of goiters, depending on the chosen definition. Treatment modalities of substernal goiter might involve some difficulties, and therefore meticulous management should be performed. Surgery is indicated as the disorder of substernal goiter became or already symptomatic, such compressive symptoms, venous congestion, tracheal deviation and/or stenosis, potential airway compromise, hyperthyroidism, and on the condition of suspicion for the malignancy on the basis of evaluation for fine-needle aspiration (FNA) cytology of the nodules within the thyroid gland [5,6].

The Bethesda System for Reporting Thyroid Cytopathology (TBSRTC), a 6-diagnostic-category system which was established by multidisciplinary formulation, was propounded at the National Cancer Institute (NCI) Thyroid Fine Needle Aspiration State of the Art and Science Conference held in Bethesda, MD, 2007. TBSRTC is at present the most used and accepted reporting system for reporting FNA cytology worldwide and its usage also has been endorsed by the 2015 American Thyroid Association (ATA) management guidelines for adult patients with thyroid nodules and differentiated thyroid cancer. In addition, Cibas and Ali reported ‘The 2017 Bethesda System for Reporting Thyroid Cytopathology’ which offers the utilization of unique term instead of the synonymous terms for the distinct category and emphasizes the updated malignancy risks based on new (post-2010) data and the recent term, non-invasive follicular thyroid neoplasm with papillary-like nuclear features (NIFTP), previously known non-invasive encapsulated follicular variant of papillary thyroid carcinoma (EFVPTC), exhibiting an indolent clinical behavior regarding invasive FVPTC [7-13]. 

The thyroid nodules with indeterminate cytology, particularly two of them, atypia of undetermined significance/follicular lesion of undetermined significance (AUS/FLUS), The Bethesda System for Reporting Thyroid Cytopathology (TBSRTC) III and follicular neoplasm/suspicious for follicular neoplasm (FN/SFN), TBSRTC IV, with the risk of malignancy (ROM) of 5% to 15% and 15% to 30%, respectively, is also a worthy category in this situation and management of the present technical report, substernal multinodular goiter. Of note, it has still been a challenging situation, for which some non-invasive applications such as elastography and molecular studies have still been debated in Endocrine Pathology, Endocrine Surgery, and Thyroidology [7-12].

The majority of the cases, over 90%, Martin et al. [1]; 93.5%, Testini et al. [14], can be treated through a Kocher's transverse collar incision while the remaining minority has been reported as might be required a partial or complete median sternotomy or even the thoracotomy. To this end, the former is usually recommended as the inferior border of the gland end up with the innominate vein, most proximally, and the latter as harboring the conditions, such as extending beyond the brachiocephalic vein towards the arcus aorta, the involvement of the posterior mediastinum, superior vena cava obstruction, recurrency, malignancy with local involvement, and emergent airway obstruction. Principally, multiplanar cross-sectional imaging modalities (computed tomography with contrast or magnetic resonance imaging) are not usually an integral part of preoperative thyroid surgical evaluation. However, they are recommended by ATA management guidelines selectively in, tumor invasion and bulky, inferiorly or posteriorly located gland and/or lymph nodes, in order of better characterization or when ultrasound expertise is not available [5,6].

Accomplishing meticulous hemostasis is essential to avoid serious complications yet the thyroid gland has been stated as a highly vascularized organ. Onwards of the recent introduction of various surgical energy devices such as LSJ vessel sealing systems and harmonic devices in order for the procedures of thyroidectomy, physicians have encountered some propounding advantages [15-17]. As such, some authors asserted that utilization of most used energy-based devices: (i) Ultrasonic Harmonic Focus Scalpel (HF), (ii) LSJ, and (iii) Thunderbeat Open Fine Jaw (TB) are effective and even safe in both hemostasis and dissection nearby the course of the inferior laryngeal nerve and even the suspensory Ligament of Berry. LSJ has been declared as utilizing advanced bipolar energy, attains hemostasis via emaciating elastin and collagen bundles in the vessel wall, rearranging them into a permanent plastic-like autologous seal structure that can seal the vessels up to seven mm in diameter [18,19]. In addition, the thyroidectomy with the relevant surgical collar procedure via intermittent or continuous intraoperative neural monitoring, IIONM, CIONM, particularly in the management of these kinds of gland pathologies have been recommended [5,20].

## Conclusions

As a matter of fact that our case was harboring a symptomatic substernal multinodular goiter, living in an iodine deficiency area, without extending beyond the left innominate vein. Herein, we performed the total thyroidectomy with transverse collar incision by using LSJ via splitting the superior thyroid arteries and veins separately. Of note, the superficial vascular layer of the Ligament of Berry was dissected and divided meticulously, exposing the RLN lying on and also lateral to the underlying, fibrous Ligament of Berry, that is, the true Ligament of Berry. To this end, we would recommend a complete sutureless thyroidectomy for substernal goiter as dividing and dissecting the superior thyroid arteries and veins separately and exploring the true Ligament of Berry with its safe interrelation to the RLN, painstakingly.
